# Genotypic diversity and clinical outcome of cryptococcosis in renal transplant recipients in Brazil

**DOI:** 10.1080/22221751.2018.1562849

**Published:** 2019-01-21

**Authors:** Vinicius Ponzio, Yuan Chen, Anderson Messias Rodrigues, Jennifer L. Tenor, Dena L. Toffaletti, José Osmar Medina-Pestana, Arnaldo Lopes Colombo, John R. Perfect

**Affiliations:** a Department of Medicine, Division of Infectious Diseases, Escola Paulista de Medicina, Universidade Federal de São Paulo (UNIFESP), São Paulo, Brazil; b Division of Infectious Disease, Department of Medicine, Duke University School of Medicine, Durham, NC, USA; c Laboratory of Emerging Fungal Pathogens, Department of Microbiology, Immunology and Parasitology, Universidade Federal de São Paulo (UNIFESP), São Paulo, Brazil; d Hospital do Rim Oswaldo Ramos Foundation, Discipline of Nephrology, Universidade Federal de São Paulo (UNIFESP), São Paulo, Brazil

**Keywords:** *Cryptococcus neoformans*, *Cryptococcus gattii*, molecular type, renal transplantation, genotypic diversity

## Abstract

Genotypic diversity and fluconazole susceptibility of 82 *Cryptococcus neoformans* and *Cryptococcus gattii* isolates from 60 renal transplant recipients in Brazil were characterized. Clinical characteristics of the patients and prognostic factors were analysed. Seventy-two (87.8%) isolates were *C. neoformans* and 10 (12.2%) were *C. gattii*. VNI was the most common molecular type (40 cases; 66.7%), followed by VNII (9 cases; 15%), VGII (6 cases; 10%), VNB (4 cases; 6.7%) and VNI/II (1 case; 1.7%). The isolates showed a high genetic diversity in the haplotype network and six new sequence types were described, most of them for VNB. There was a bias towards skin involvement in the non-VNI population (*P* = .012). VGII isolates exhibited higher fluconazole minimum inhibitory concentrations compared to *C. neoformans* isolates (*P* = 0.008). The 30-day mortality rate was 38.3%, and it was significantly associated with fungemia and absence of headache. Patients infected with VGII had a high mortality rate at 90 days (66.7%). A variety of molecular types produce disease in renal transplant recipients in Brazil and highlighted by VGII and VNB. We report the clinical appearance and impact of the molecular type, fluconazole susceptibility of the isolates, and clinical characteristics on patient outcome in this population.

## Introduction

Cryptococcosis is a life-threatening invasive fungal disease caused by the encapsulated yeasts, *Cryptococcus neoformans* and *Cryptococcus gattii* [1]. *C. neoformans* has a worldwide distribution affecting predominantly individuals with impaired cell-mediated immunity and *C. gattii* has a more limited environmental distribution and a higher percentage of disease within apparently normal hosts [1]. In renal transplant recipients, cryptococcosis is recognized as the second most common invasive fungal infection, with incidence rates ranging from 0.3% to 5.8% and overall mortality rates as high as 20–50% [2–11].

The nomenclature of *C. neoformans/C. gattii* species complexes is continuing to evolve under molecular evidences [12,13]. However, as a starting point, cryptococcosis is caused primarily by two species *C. neoformans* and *C. gattii* and currently these species can be further divided into ten molecular siblings known as VNI, VNII, VNB, VNIII, VNIV, VGI, VGII, VGIII and VGIV [12] with a possible new molecular type designated VGV. The most widely utilized sequence-based genotyping method for the molecular identification of these complexes has been multilocus sequence typing (MLST). This method is robust and portable between laboratories [14,15].

Clinical comparative studies and understandings between different cryptococcal molecular types are still in their infancy and remain controversial whether or not these different molecular types represent specific characteristics in terms of clinical manifestations or attributable mortality rates [13,16–18]. Furthermore, most data related to strain distribution of *C. neoformans* and *C. gattii* species complexes in the transplant recipient relies on small series and case report [5,19–23].

The purpose of our study was to characterize the molecular types of *C. neoformans* and *C. gattii* isolated and to assess the clinical outcome of cryptococcosis and their molecular types in patients undergoing renal transplantation throughout Brazil. Interestingly, Brazil represents an environment with a diverse number of cryptococcal molecular types and likely has the most cryptococcal strain diversity of any country practising routine kidney transplantation [24,25].

## Results

### Clinical characteristics

We enrolled a total of 60 renal transplant recipients followed for a median period of 4 months (0 days to 11 years). One patient had received a liver transplant allograft one year before kidney transplantation and another patient had undergone simultaneous pancreas-kidney transplantation. The clinical characteristics are outlined in Table 1.

**Table 1.** Demographic and clinical characteristics of 60 renal transplant recipients infected by *C. neoformans/C. gattii* species complexes.

Characteristics	Value, % (no. of patients) *n* = 60
Age, average years (range)	49 (21–71)
Male	63.3 (38)
Ethnicity	
White	60 (36)
Non-white	40 (24)
Retransplant^a^	6.7 (4)
Donor type	
Deceased	60 (36)
Living	40 (24)
Immunosuppressive induction therapy	40 (24)
Immunosuppressive agents received^b^	
Prednisone	100 (60)
Tacrolimus	56.7 (34)
Mycophenolic acid^c^	55 (33)
Azathioprine	26.7 (16)
Cyclosporine A	18.3 (11)
Rapamycin	8.3 (5)
Prior rejection	40 (24)
Diabetes mellitus	26.7 (16)
Active cytomegalovirus infection	25 (15)
Hepatitis C infection	18.3 (11)
Time to onset of infection after transplant, average months (range)	30.5 (13 days to 17 years)
Sites of involvement	
CNS	83.3 (50)
Pulmonary	50 (30)
Skin, soft-tissue, or osteoarticular	20 (12)
Fungemia	38.3 (23)
Disseminated infection^d^	63.3 (38)
Renal failure at baseline^e^	56.7 (34)
Serum cryptococcal antigen titre, median (range)^f^	1:1024 (0–1:1024)
Change in immunosuppression at diagnosis^g^	78.6 (44)
Antifungal therapy	
Amphotericin B alone	60 (36)
Amphotericin B + 5FC	18.1 (11)
Amphotericin B + fluconazole	8.3 (5)
Fluconazole	3.3 (2)
None	10 (6)
Mortality at 90 days	45 (27)

Note: CNS, central nervous system; 5FC, 5-flucytosine.

^a^Indicates prior receipt of a renal transplant.

^b^Immunosuppressive agent that remained unchanged within 3 months of the onset of cryptococcosis.

^c^Includes mycophenolate mofetil or mycophenolate sodium.

^d^Defined as the involvement of at least two noncontiguous organ systems or the presence of fungemia.

^e^Indicates creatinine ≥2 mg dL^−1^ at the time of diagnosis of infection.

^f^Data available for 35 patients.

^g^Data available for 56 patients.

### Molecular characterization and clinical associations

**Figure 1.**
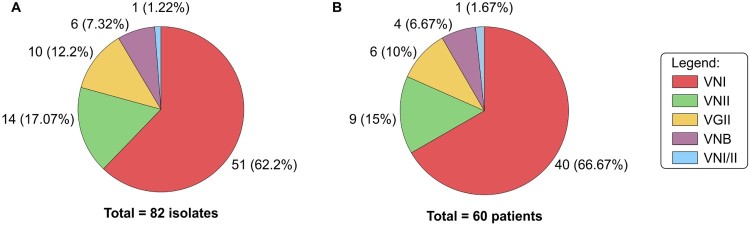
Molecular type distribution of 82 clinical isolates of *C. neoformans/C. gattii* species complexes (A) cultured from 60 renal transplants recipients (B).

We collected 82 isolates of *C. neoformans/C. gattii* species complexes from 60 renal transplant recipients. Ten (12.2%) isolates were identified as *C. gattii* and 72 (87.8%) isolates as *C. neoformans* (Figure 1(A)). Forty-seven isolates were from cerebrospinal fluid (CSF), 23 from blood, 6 from pulmonary secretions, 5 from skin biopsy and 1 from urine. The distribution of different molecular types among the patients is depicted in Figure 1. The most common molecular type was VNI (51 isolates from 40 patients). For 20 episodes on which there were more than one isolate per patient, all but two exhibited similar molecular type within the same episode. In both patients infected by different molecular types we isolated VNI followed by VNII separated by 15 days in CSF or 44 days in blood. These two cases had their findings confirmed by three independent assays yielding the same results. In our entire cohort, only one isolate was diploid based on flow cytometry (VNI/VNII).

A total of 81 isolates were analysed by MLST and compared to MLST database. Nine allele types have been identified for the *URA5* locus (1 new), 10 for *LAC1* (1 new), 9 *SOD1* (1 new), 14 for *IGS1* region (3 new), 10 for *CAP59* (1 new), 11 for *PLB1* and 8 for *GPD1* (1 new). Based on the combined analysis of the 7 MLST loci for *C. neoformans*, 20 sequence types (ST) were found, in which 6 were new (ST581, ST587, ST588, ST589, ST590 and ST591). Of those new ST, four were VNB molecular type and two VNII (Supplementary Table 1).

**Figure 2.**
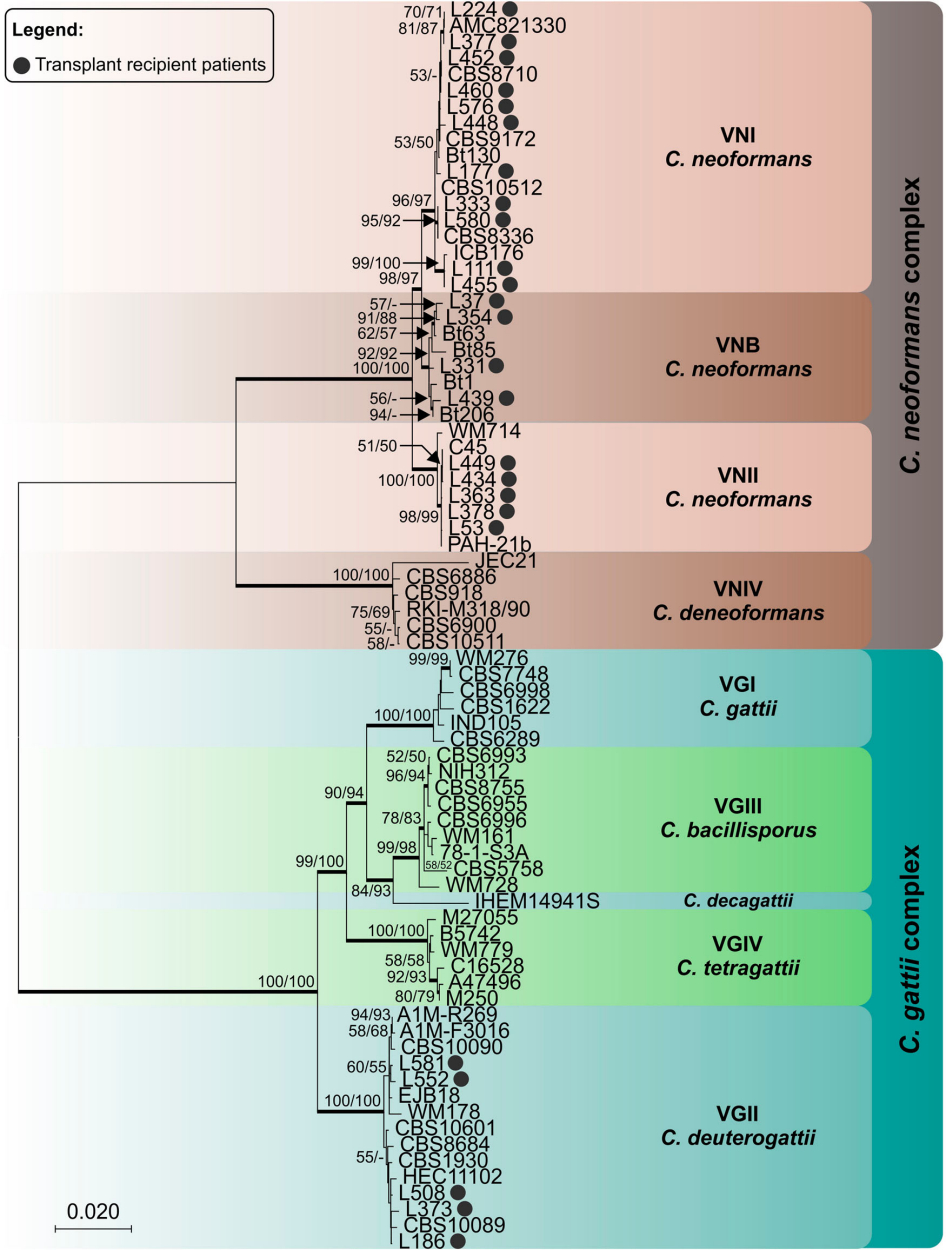
Phylogenetic relationships as inferred from a maximum likelihood analysis of *CAP59*, *LAC1*, *PLB1*, *SOD1*, *URA5*, *TEF1* and *IGS1* sequences from 82 strains of *C. neoformans* and *C. gattii* from transplant patients and 63 reference strains, covering the main molecular types described. The numbers close to the branches represent indices of support (maximum likelihood/neighbor-joining) based on 1000 bootstrap replications. The branches with bootstrap support higher than 70% are indicated in bold.

Figure 2 shows the phylogenetic tree of one representative sequence for each haplotype found for these transplant recipient isolates in relationship to reference strains. Our VGII isolates did not cluster with VGIIa, VGIIb or VGIIc.

**Figure 3.**
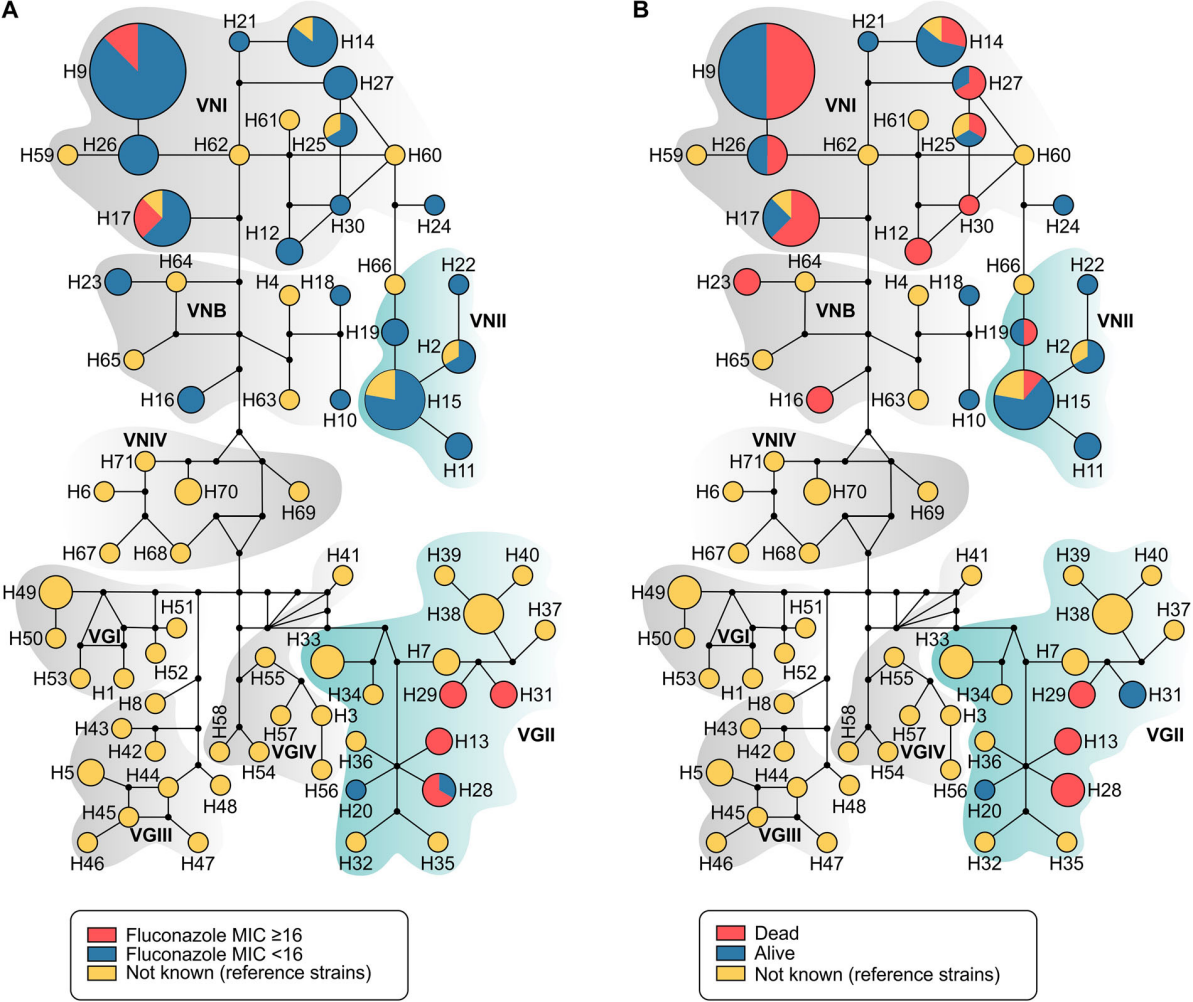
Median-joining haplotype network of 144 isolates of *C. neoformans*/*C. gattii* species complexes (81 isolates originated in this study in addition to 63 reference strains recovered from literature), covering all the concatenated loci *CAP59*, *LAC1*, *PLB1*, *SOD1*, *URA5*, *TEF1* and *IGS1* sequences. The isolates are coded, and their frequencies are represented by (A) fluconazole MIC ≥ than 16 mg l^−1^ from transplant recipients isolates or (B) 90-days mortality of transplant recipients. The size of the circumference is proportional to the haplotype frequency. The black dots (median vectors) represent unsampled or extinct haplotypes in the population. Further information about isolate source and GenBank accession number can be found in the Supplementary Table 1.

For haplotype analyses we employed a dataset of 144 sequences (7 loci) from *C. neoformans/C. gattii* species complexes, being 81 generated in this study and 63 recovered from GenBank (Supplementary Table 1). Haplotype and nucleotide diversities were high in our overall dataset (number of haplotypes = 71; Hd = 0.961; π = 0.07003) and also specifically within our 81 transplant recipient isolates (Hd = 0.890; π = 0.03379). Remarkably, 24 out of 71 different haplotypes found in the full dataset for *C. neoformans/C. gattii* species complexes originated from transplant recipients. Only four haplotypes (H14, H15, H17 and H25) were identical to reference strains, revealing the high diversity of our isolates (Figure 3; Supplementary Table 1).

Regarding clinical correlations, patients infected by *C. gattii* were more likely to be a retransplant than those infected with *C. neoformans* (*P* = .046) (Table 2). Furthermore, patients infected with non-VNI molecular types were significantly more likely to have skin involvement than patients infected with VNI (40% vs. 10% patients, respectively; OR = 5.77; 95% CI = 1.47–22.56; *P* = .012).

**Table 2.** Comparisons of infections due to *C. neoformans* and *C. gattii* complex in 60 renal transplant recipients.

	Univariate analysis			Multivariate analysis		
Characteristics (N°. patients) *n* = 60	*C. neoformans* infection % (N^o^. of patients) *n *= 54	*C. gattii* infection % (N^o^. of patients) *n* = 6	*P-*value	OR	95% CI	*P-*value
Age, average years (range)	49 (21–69)	50.5 (32–71)	0.796			
Male	66.7 (36)	33.3 (2)	0.179			
White	61.1 (33)	33.3 (2)	0.223			
Living in the capital before infection	27.8 (15)	6.3 (1)	1.0			
Northeast of Brazil as place of birth	31.5 (17)	50 (3)	0.390			
**Retransplant**^a^	**3.7 (2)**	**33.3 (2)**	**0.046**			
Deceased donor type	57.4 (31)	83.3 (5)	0.387			
Induction immunosuppressive therapy	38.9 (21)	50 (3)	0.675			
Prior rejection	38.9 (21)	50 (3)	0.675			
Cytomegalovirus infection	25.9 (14)	16.7 (1)	1.0			
Time to onset of infection after transplant, average months (range)	30 (13 days–17 years)	37.5 (188 days–7 years)	0.667			
Sites of involvement						
CNS (56)	90.6 (48)	66.7 (2)	0.293			
Pulmonary (59)	48.1 (26)	80 (4)	0.353			
Skin, soft-tissue, or osteoarticular	16.7 (9)	50 (3)	0.088			
Fungemia	35.2 (19)	66.7 (4)	0.191			
Disseminated infection^b^	61.1 (33)	83.3 (5)	0.4			
Renal failure at baseline^c^	53.7 (29)	83.3 (5)	0.221			
CNS image abnormality (37)	20 (7)	0 (0)	1.0			
Diffuse infiltrate in lung image (27)	37.5 (9)	0 (0)	0.529			
Serum cryptococcal antigen titre ≥ 1:512 (31)	57.1 (16)	100 (3)	0.265			
Mean duration of hospitalization, ±SD (range), days	36.8 ± 38.19 (1–245)	9.33 ± 11.89 (8–33)	0.087			
Duration of antifungal induction therapy, average days (range) (54)	26.3 (1–219)	16 (4–28)	0.504			
Total duration of antifungal therapy, average days (range) (51)	115 (1–635)	288.5 (193–384)	0.453			
**Fluconazole MIC** ≥ **16 mg l^−1^**	**7.4 (4)**	**66.7 (4)**	**0.002**	**12.62**	**1.97–80.99**	**0.008**
Mortality at 90 days	42.6 (23)	66.7 (4)	0.394			

Note: CNS, central nervous system; SD, standard deviation; MIC, minimum inhibitory concentration.

^a^Indicates prior receipt of a renal transplant.

^b^Defined as the involvement of at least two noncontiguous organ systems or the presence of fungemia.

^c^Indicates creatinine ≥2 mg dL^−1^ at the time of diagnosis of infection.

Fluconazole minimum inhibitory concentration (MIC) distributions for *C. neoformans* and *C. gattii* isolates varied as depicted in Table 3. Modal MICs were 2–16 mg l^−1^, with the higher mode for VGII (16 mg l^−1^) and lowest mode for VNII (2 mg l^−1^). For example, 13 out of 82 (15.85%) isolates exhibited fluconazole MIC values that were ≥16 mg l^−1^, in which 8 (61.54%) were represented by molecular type VGII (Figure 3(A)). By multivariate analysis, fluconazole MIC values were higher in patients infected by *C. gattii* when compared to *C. neoformans* (OR = 12.62; 95% CI = 1.97–80.99; *P* = .008) (Table 2).

**Table 3.** Fluconazole MIC distribution for 82 isolates of *C. neoformans/C. gattii* species complexes tested.

						No. of isolates for which the MIC (mg l^−1^) was^a^:									
Molecular type or specie	Number of isolates	Mean mg l^−1^	Interval mg l^−1^	MIC50 mg l^−1^	MIC90 mg l^−1^	≤0.12	0.25	0.5	1	2	4	8	16	32	≥64
All isolates	82	8.70	0.25–64	8	16		1		3	11	20	**34**	9	2	2
*C. neformans*	72	6.18	0.25–16	8	8		1		3	10	19	**33**	5		
VNI	51	7.22	0.25–16	8	8		1			2	15	**28**	5		
VNII	14	2.50	1–8	2	4				3	**8**	2	1			
VNB	6	6.86	4–8	8	8						2	**4**			
VNI/II	1										1				
*C. gattii –* VGII	10	25.82	4–64	16	64						1	1	**4**	2	2

Note: MIC, minimum inhibitory concentration.

^a^The modal MIC for each distribution is underlined.

### Mortality and prognostic factors

**Figure 4.**
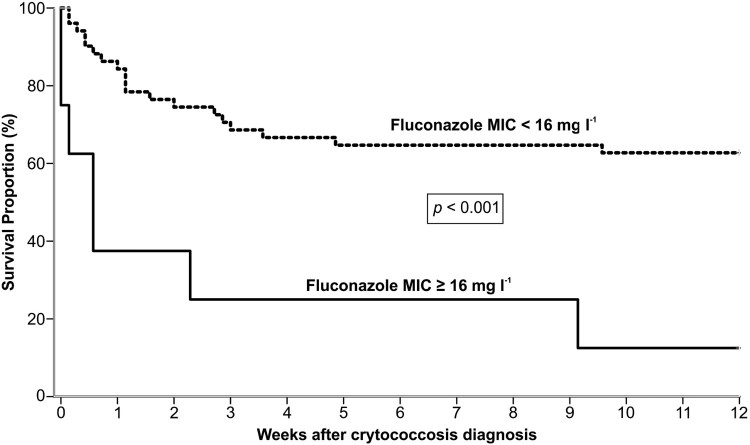
Kaplan–Meier analysis of 12 weeks survival of 60 renal transplant recipient infected by *C. neoformans*/*C. gattii* species complexes according to fluconazole MIC ≥ 16 mg l^−1^ (*n* = 8) or MIC < 16 mg l^−1^ (*n* = 52).

In our study, 23 out of 60 patients (38.3%) died within 30 days after diagnosis of cryptococcosis, and 4 more (total of 45%) died within 90 days. Finally, the overall mortality rate in our study group was 61.7% (37/60), over the entire follow-up period. Based on the univariate analysis, the following factors were significantly associated with high mortality at 30 days: induction immunosuppressive therapy, deceased donor, receipt of a calcineurin-inhibitor agent, pulmonary infection, fungemia, somnolence and absence of headache at admission, CSF antigen titre >1:512 at diagnosis, positive pulmonary culture, discontinuation of tacrolimus after infection, graft loss within 30 days and patients infected with isolates showing MIC ≥ 16 mg l^−1^. After multivariate regression analysis, the factors independently associated with 30-day mortality were fungemia (OR = 3.79; *p* = .044) and absence of headache (OR = 0.13; *p* = .001) (Table 4). Most of our patients infected by VGII died within 90 days after cryptococcosis onset. In contrast, there was high survival rate of patients infected by VNII haplotype (Figure 3(B)). Furthermore, the probability of survival after 12 weeks of cryptococcosis was significantly lower in patients infected with isolates exhibiting fluconazole MICs ≥ 16 mg l^−1^compared to those with MICs < 16 mg l^−1^ (*P* < .001, log rank test) (Figure 4).

**Table 4.** Variables associated with 30-day mortality after cryptococcosis in 60 renal transplant recipients.

	Univariate analysis			Multivariate analysis		
	Survival, %	Death, %				
Variables (N°. patients) *n* = 60	*n* = 37	*n* = 23	*P-*value	OR	95% CI	*P-*value
Age, mean, ±SD, years (60)	48.1 ± 12.51	49.6 ± 12.94	0.657			
Male (38)	56.8	73.9	0.180			
Induction immunosuppressive therapy (24)	27	60.9	**0.009**			
Deceased donor (36)	48.6	78.2	**0.023**			
Prior rejection (24)	43.2	34.8	0.515			
Cytomegalovirus infection^a^ (7)	13.5	8.7	0.697			
Receipt of a calcineurin-inhibitor agent^b^ (45)	64.9	91.3	**0.021**			
Receipt of a tacrolimus^b^ (34)	45.9	73.9	**0.034**			
Duration of symptoms before diagnosis, mean, ±SD (56)	35.4 ± 42.69	29.2 ± 78.74	0.705			
Time to diagnose after admission, mean, ±SD (57)	4.7 ± 9.09	3.6 ± 4.65	0.585			
Sites of involvement						
CNS^c^ (50)	89.2	89.5	1			
Pulmonary^d^ (30)	40.5	68.2	**0.04**			
Skin or soft-tissue (12)	24.3	13	0.34			
Disseminated infection (38)	54.1	78.3	0.059			
**Fungemia (23)**	**18.9**	**69.6**	**<0.001**	**3.79**	**1.03–13.91**	**0.044**
*C. gattii* infection (6)	5.4	17.4	0.191			
Non-VNI genotype (20)	32.4	34.5	0.851			
Creatinine at admission ≥2 mg dL^−1^ (34)	51.4	65.2	0.292			
Somnolence at admission^e^ (19)	27.3	58.8	**0.029**			
Confusion at admission^e^ (19)	30.3	52.9	0.118			
**Headache at admission**^e^**(30)**	**72.7**	**35.3**	**0.01**	**0.13**	**0.04–0.42**	**0.001**
Intracranial hypertension^f^ (16)	48.1	33.3	0.7			
Respiratory failure^b,g^ (7)	6.7	40	0.08			
CSF cell count^b^, mean, ±SD, mm^3^ (42)	138.5 ± 250.04	101.1 ± 92.8	0.664			
CSF glucose ratio^b^, mean, ±SD, mg dL^−1^ (41)	47.3 ± 24.63	39.2 ± 31.89	0.420			
Positive CSF India ink^h^ (23)	42.4	64.3	0.170			
CSF antigen titre >1:512^i^ (24)	43.3	84.6	**0.012**			
Positive pulmonary culture^j^ (9)	33.3	100	**0.021**			
AMBd as primary therapy^k^ (53)	97.3	100	1			
Combination therapy^k^ (16)	24.3	41.2	0.208			
Change in immunosuppressive regime after infection^c^ (44)	80.6	75	0.737			
Discontinuation of tacrolimus after infection^l^ (24)	41.4	80	**0.025**			
Graft loss within 30 days^m^ (7)	13.5	100	**0.028**			
Fluconazole MIC ≥ 16 mg l^−1^ (8)	25	75	**0.045**			

Note: OR, odds ratio; CI, confidence interval; SD, standard deviation; CNS, central nervous system; CSF, cerebrospinal fluid; AMBd, amphotericin B deoxycholate; MIC, minimum inhibitory concentration.

^a^Infection occurring within 6 months of the onset of cryptococcosis.

^b^At cryptococcosis diagnosis.

^c^Data was available for 56 patients.

^d^Data was available for 59 patients.

^e^Data was available for 50 patients.

^f^Data was available for 36 patients.

^g^Data was available for 30 patients.

^h^Data was available for 47 patients.

^i^Data was available for 43 patients.

^j^Data was available for 13 patients.

^k^Data was available for 54 patients, at least 2 consecutive days of the same antifungal therapy.

^l^Data was available for 44 patients.

^m^Data was available for 39 patients.

## Discussion

Our retrospective analysis describes 60 cases of cryptococcosis among renal transplant recipients documented during a period of 26 years. The patients presented with high rates of fungemia (38.3%), common disseminated infection (63.3%), and substantial 90-day mortality (45%). These data are comparable to some prior studies evaluating cryptococcosis in renal transplant recipients [3,5–8,26,27]. However, lower rates of mortality have been recently reported among all types of solid transplant recipients in high resource-available countries [2,9–11,26–28]. Furthermore, in our cohort of transplant recipients infected by *C. gattii*, there was an impressive 66.7% mortality rate within 90 days. Similarly, other investigators have found high rates of dissemination (63.6%) and a high 90-day mortality (36%) for solid organ transplant (SOT) recipients infected by *C. gattii* [19]. These observations emphasize the potential need for clinicians to know the identification of the species of *Cryptococcus* in an individual transplant recipient infection for prognostic determination and possibly management alterations.

We observed clinically that fungemia and absence of headache were significantly correlated with decreased survival in our kidney transplant recipients. These characteristics are likely to identify a patient with a high burden of yeasts and/or late diagnosis. These clinical characteristics have been found to be poor prognostic features in other patient populations [29,30].

In Brazil, most cryptococcal molecular types in infections are represented. Indeed, MLST and whole-genome based population analyses suggest that Brazil could be a global centre for diversity of *C. neoformans*/*C. gattii* species complexes and even a location for species origin [21,25,31–33]. However, studies addressing the distribution of specific molecular types of *Cryptococcus* infecting transplant recipients remain scarce worldwide. In the setting of SOT, one study in China revealed 9 cases of VNI and a recent study described 10 strains of molecular type VGII and one of VGI from the Pacific Northwest of USA outbreak. In this last study, 18% of the strains in transplant recipients were *C. gattii* [5,19]. We found in our cohort of renal transplant recipients that *C. gattii* represented 12% of isolates (VGII). While VNI was the most common molecular type infecting our patients (66.7%), some genotypic diversity with *C. neoformans* was also found as we detected 14 VNII and 6 VNB isolates. The molecular predominance of VGII may represent either a specific tropism for transplant recipients and/or increased resistance to tacrolimus/cyclosporine or more likely higher exposure to VGII in the environment over the other VG molecular types. A higher frequency of *C. gattii* disease observed in retransplantation recipients supports the hypothesis that cryptococcal disease of immunocompromised hosts with *C. gattii* may be more a primary disease while *C. neoformans* disease more frequently represents secondary or reactivation disease [4].

Surprisingly we found VNB isolates of *C. neoformans* in this Brazilian cohort, a molecular type that was considered to be geographically restricted to Africa [34]. However, recently, almost a dozen clinical and environmental isolates from Italy, Portugal, China and Brazil have been identified as VNB strains [20,35–39]. In fact, a recent study comparing Brazilian VNB isolates from transplant recipients with African isolates showed a high diversity within these isolates, except for one isolate from Brazil that nested deeply within the African clade on the phylogeny tree [31]. This isolate was recovered from a mulatto patient living in São Paulo and might have corresponded to a recent migration event. However, VNB isolates as a whole may support the fact that their geographical niche was separated during the Pangea period in which continents split [40].

The Next Generation MLST (NGMLST) methodology and primers that we used were not able to properly sequence the *GPD1* locus for VGII molecular type as previously reported [14]. Another issue for our genotyping analysis was our inability to precisely separate our VGII isolates into three distinct clonal lineages (VGIIa, VGIIb and VGIIc). MLST and whole-population genome studies comparing the VGII outbreak strains with VGII isolates from other regions showed that isolates, especially from south of Brazil, have not clustered with any of the three specific lineages [25,33,41].

The clinical impact of different cryptococcal molecular types on patient outcome is still unknown. Controversial results have been generated in the correlation between molecular types and virulence based on experimental studies [13,16,18] and clinical data [17,20,42,43]. We failed to demonstrate substantial differences in clinical presentation or outcome by molecular types, except that non-VNI molecular strains were significantly more likely to show skin involvement than patients infected by VNI strains. There were also no significant differences in outcomes between the *C. neoformans* and *C. gattii* infections with the important caveat that there were fewer *C. gattii* strains and all were VGII. Consequently, the statistical power to detect differences in our study is limited. However, these initial observations support the hypothesis that virulence is not consistently associated with a single major molecular type or subtype but may be more related to the distinct properties of individual isolates.

Fluconazole susceptibility profile is a potentially important clinical issue but its relevance in terms of precisely determining prognosis is still controversial, especially in the setting of the transplant population [5,19,44,45]. In general, direct correlation between species type and antifungal susceptibility has varied [18,21,46–48]. Our VGII isolates showed high fluconazole MICs. Furthermore, in our study, infection by *C. neoformans*/*C. gattii* species complex isolates exhibiting high fluconazole MICs did correlate with a worse patient survival rate. However, the precise clinical correlation of *in vitro* antifungal susceptibility testing of *Cryptococcus* and break points remains to be further defined [49–53].

In conclusion, cryptococcosis in the setting of SOT in less-resourced countries should be considered a life-threatening fungal disease with substantial mortality rates. The high mortality is probably due to late diagnosis, suboptimal antifungal therapy, including lack of 5FC, as well as the virulence properties of these fungi in SOT. In fact, this transplant population in Brazil represents a relatively uniform host with high rates of mortality for cryptococcosis that may allow for the detection and characterization of particularly virulent strains. With this diversity of strains and a similar high-risk host population for mortality, further examination of these transplant cohorts in Brazil and their fungal strains may give us signals into how these yeasts specifically cause aggressive disease.

## Materials and methods

### Isolates

We evaluated all *C. neoformans* and *C. gattii* strains isolated from patients who underwent renal transplantation and subsequently developed cryptococcosis from 1987 to 2013 at São Paulo Hospital or the Kidney Hospital in São Paulo. The isolates were stored in cryopreservative medium at −70°C with Yeast Peptone Dextrose (YPD) and 20% glycerol at the Special Mycology Laboratory – Federal University of São Paulo.

### Clinical data and definitions

The following variables were collected from the medical records using a standard clinical report form: demographic data, complications prior to and after transplantation, immunosuppressive regimens, antifungal therapy, and laboratory data. Disseminated cryptococcosis was defined as the involvement of at least two noncontiguous organ systems or the presence of fungemia [6]. Primary therapy was defined as the first systemic antifungal regimen administered for at least two consecutive days [54]. The study was approved by the local ethic committee (UNIFESP Number: 318847, 2013).

### Genotyping

A single colony of each isolate was isolated, grown in YPD broth and frozen at −70°C with glycerol. To isolate genomic DNA, the isolates were streaked from these frozen stocks onto fresh YPD agar, grown for 2–4 days. Next, several colonies of each sample were used to extract and purify the genomic DNA, using the MasterPure Yeast DNA Purification Kit (Epicentre Biotechnologies, Madison, WI, USA). We analysed the sequences from eight loci, including seven standard gene regions (*CAP59*, *LAC1*, *PLB1*, *SOD1*, *URA5*, *TEF1*, *GPD1*) and the intergenic spacer region 1 (*IGS1*) of the nuclear ribosomal RNA gene. The primer sequences and the preparation of the sequencing libraries were based on the NGMLST method previously described and the raw sequencing data was processed using MLSTEZ [14]. The allele type of each generated consensus sequence for every locus was determined using the MLST database (http://mlst.mycologylab.org).

The phylogenetic analysis was performed using MEGA 7.0 [55]. The evolutionary relationships, with 1000 bootstrap replicates of the concatenated nucleotide sequences, were inferred using maximum likelihood and the neighbor-joining methods [56]. Major molecular types were confirmed according to phylogenetic clustering with reference strains. Evolutionary relationships at the intraspecific level were evaluated using haplotype networks in order to visualize differences and diversity among isolates. Haplotype and nucleotide diversities were estimated using DNAsp v5.0 [57]. Median-joining networks for the dataset were obtained and visualized using the software Network 5.0 (Fluxus Technology). Flow cytometry was used to check ploidy of the hybrid isolate [58].

### Antifungal susceptibility testing

The determination of MICs for fluconazole was performed using the CLSI broth microdilution assay, according to the M27-A3 document [59]. *Candida parapsilosis* ATCC 22019 and *Candida krusei* ATCC 6258 were included as quality controls. Assays were performed in RPMI with endpoints read after 72 h at 35°C. The MIC was defined as the lowest concentration that produced 50% growth inhibition compared with the drug-free growth control. The interpretation of MIC values was based on epidemiological cutoff values [60].

### Statistical analysis

Continuous data were presented as either the mean ± SD or the median and range, and categorical data were presented as proportions. Univariate analyses were performed to compare categorical variables using either the Chi-square or Fisher's exact test as appropriate and continuous variables by Student's *t*-test. Variables whose univariate test result had a *P*-value <.1 were considered candidates for the multivariate model. Binary logistic models were generated using forward stepwise selection for factors associated with death and comparisons between molecular types and species. All analyses were performed using SPSS software for Windows, version 22 (SPSS, Chicago, IL). A value of *P* ≤ .05 was considered statistically significant.

### Data availability

The sequences of all newly identified allele types have been submitted to the MLST database (http://mlst.mycologylab.org) and GenBank (https://www.ncbi.nlm.nih.gov/genbank/).
